# Antalarmin

**DOI:** 10.1107/S1600536808023775

**Published:** 2008-07-31

**Authors:** Stephen R. Slauson, John M. Rimoldi, Frank R. Fronczek

**Affiliations:** a University of Mississippi, Department of Medicinal Chemistry, University, MS 38677, USA; b Department of Chemistry, Louisiana State University, Baton Rouge, LA 70803-1804, USA

## Abstract

In the mol­ecule of the title compund [systematic name: *N*-butyl-*N*-ethyl-2,5,6-trimethyl-7-(2,4,6-trimethyl­phen­yl)-7*H*-pyrrolo[2,3-*d*]pyrimidin-4-amine], C_24_H_34_N_4_, the pyrrolopy­rimidine ring system is nearly planar, its five- and six-membered rings forming a dihedral angle of 5.3 (2)°. The benzene ring is nearly orthogonal to the central ring system. The N atom carrying the ethyl and *n*-butyl groups is flattened pyramidal.

## Related literature

For related literature, see: Allen (2002 ▶); Chorvat *et al.* (1999 ▶); Chu *et al.* (2007 ▶); Dieterich *et al.* (1997 ▶); Gross *et al.* (2005 ▶); Habib *et al.* (2000 ▶); Horn *et al.* (2008 ▶); Hsin *et al.* (2002 ▶); Banić Tomišić *et al.* (2001 ▶); Rivier & Vale (1983 ▶); Steckler & Holsboer (1999 ▶); Vale *et al.* (1981 ▶); Greiner *et al.* (2002 ▶).
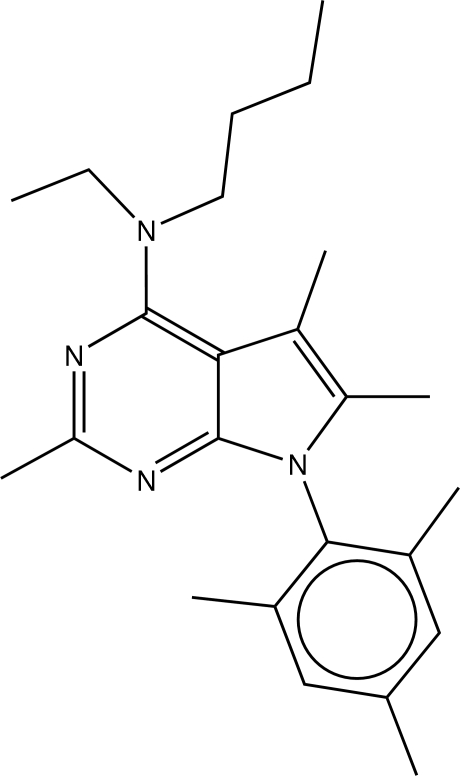


## Experimental

### 

#### Crystal data

C_24_H_34_N_4_
*M* *_r_* = 378.55
Triclinic, 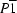

*a* = 10.2656 (10) Å
*b* = 11.0655 (11) Å
*c* = 11.5029 (12) Å
α = 63.646 (6)°
β = 85.669 (6)°
γ = 73.036 (5)°
*V* = 1117.69 (19) Å^3^
*Z* = 2
Mo *K*α radiation
μ = 0.07 mm^−1^
*T* = 150 K
0.40 × 0.37 × 0.30 mm

#### Data collection

Nonius KappaCCD diffractometer with Oxford Cryostream
Absorption correction: none
35420 measured reflections
8550 independent reflections
6916 reflections with *I* > 2σ(*I*)
*R*_int_ = 0.023

#### Refinement

*R*[*F* ^2^ > 2σ(*F* ^2^)] = 0.049
*wR*(*F* ^2^) = 0.142
*S* = 1.03
8550 reflections
262 parameters
H-atom parameters constrained
Δρ_max_ = 0.44 e Å^−3^
Δρ_min_ = −0.24 e Å^−3^

### 

Data collection: *COLLECT* (Nonius, 2000 ▶); cell refinement: *HKL* *SCALEPACK* (Otwinowski & Minor 1997 ▶); data reduction: *HKL* (Otwinowski & Minor 1997 ▶) *DENZO* and *SCALEPACK*; program(s) used to solve structure: *SIR97* (Altomare *et al.*, 1999 ▶); program(s) used to refine structure: *SHELXL97* (Sheldrick, 2008 ▶); molecular graphics: *ORTEP-3 for Windows* (Farrugia, 1997 ▶); software used to prepare material for publication: *SHELXL97*.

## Supplementary Material

Crystal structure: contains datablocks global, I. DOI: 10.1107/S1600536808023775/pv2088sup1.cif

Structure factors: contains datablocks I. DOI: 10.1107/S1600536808023775/pv2088Isup2.hkl

Additional supplementary materials:  crystallographic information; 3D view; checkCIF report

## Figures and Tables

**Fig. 1.**
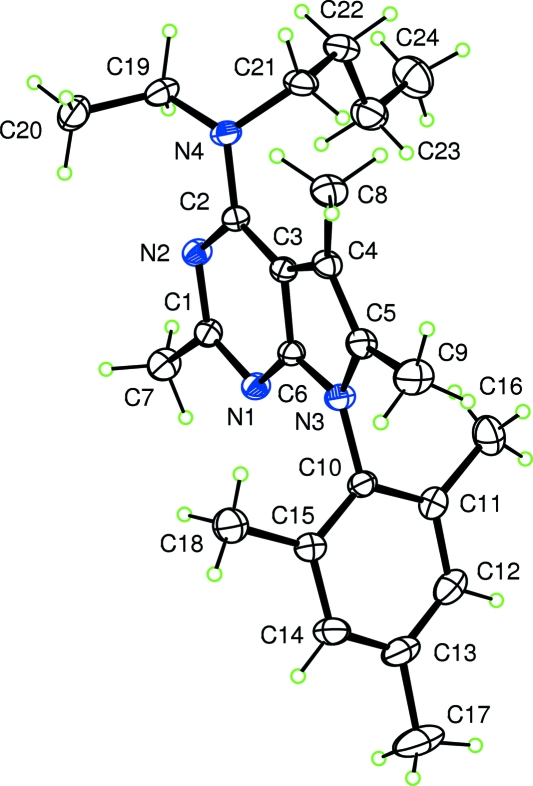
Ellipsoids at the 50% level, with H atoms having arbitrary radius.

## supplementary crystallographic information

### Comment

Corticotropin releasing factor (CRF) is a 41 amino acid hormone that has been implicated in the stress response cascade (Vale *et al.*,1981). This action is achieved by the release of adrenocorticotropic hormone (ACTH) by stimulation of the hypothalamic-pituitary-adrenal (HPA) axis (Rivier & Vale, 1983). CRF creates this release by binding to G-protein coupled receptors designated as CRF1 and CRF2 (Steckler & Holsboer, 1999). The CRF1 receptor has been designated as the subtype responsible for the physiological reaction to stress (Dieterich *et al.*, 1997). Both subtypes are found widely distributed in the central nervous system and have been correlated to numerous other responses (Hsin *et al.*, 2002).

In the search for non-peptide, selective antagonists for CRF1, antalarmin (1) has emerged as a lead candidate. Analogs have been developed to increase activity and decrease LogP, yet none have been reported as superior in overall performance (Gross *et al.*, 2005; Hsin *et al.*, 2002; Chorvat *et al.*, 1999). Antalarmin has shown anxiolytic activity in primates, as well as a reduction of self-administration of ethanol in addiction models (Habib *et al.*, 2000; Chu *et al.*, 2007). Recently, detailed toxicology has been reported indicating a favorable safety profile and suggesting that clinical trials may begin soon (Horn *et al.*, 2008).

Data collection was initially attempted at 90 K, but an apparent phase change destroyed the crystal at that temperature. Thus, data were collected at 150 K, and the structure based upon those data is presented in Fig. 1. The pyrimidine and pyrrole rings are slightly nonplanar, exhibiting maximum deviations 0.036 (1) Å (for C2) and 0.016 (1) Å (for C6), respectively. These planes form a dihedral angle of 5.3 (2)°. The phenyl group is nearly orthogonal to the pyrrolopyrimidine ring system, forming dihedral angles of 88.52 (2)° with the pyrimidine and 86.40 (3)° with the pyrrole plane. N3 is trigonal planar, lying 0.011 (1) Å from the plane defined by the three C atoms bonded to it. Amine nitrogen N4 is flattened pyramidal, lying 0.252 (1) Å from the plane of C2, C19 and C21, with C–N–C angles in the range 115.32 (7)–118.08 (7)°. The *n*-butyl group is extended except for its attachment to N, with C19—N4—C21—C22 torsion angle -54.51 (11)°.

No other pyrrolopyrimidines having substituents at N4 are found in the Cambridge Structural Database (version 5.29, Nov. 2007; Allen, 2002). The structure of a similar molecule, ICAKOM, having a cyclopentyl group at N3 and a 4-phenoxyphenyl group at C4, has been reported Banić Tomišić *et al.* (2001).

### Experimental

Synthesis of antalarmin has been reported (Greiner *et al.*, 2002). It was crystallized from acetonitrile:water to afford translucent crystals: Mp = 85.9–86.1°C; HRMS m/*z* expected: 379.2862 found: 379.2878; CHN analysis for C_24_H_34_N_4_ expected: C 76.15, H 9.05, N 14.80%; found: C 76.07, H 8.69, N 14.90%.

### Refinement

H atoms on C were placed in idealized positions with C—H distances 0.95 - 0.99 Å and thereafter treated as riding. *U*_iso_ for H was assigned as 1.2 times *U*_eq_ of the attached atoms (1.5 for methyl). A torsional parameter was refined for each methyl group.

### Crystal data

**Table:**

C_24_H_34_N_4_	*Z* = 2
*M**_r_* = 378.55	*F*_000_ = 412
Triclinic, *P*1	*D*_x_ = 1.125 Mg m^−^^3^
Hall symbol: -P 1	Mo *K*α radiation λ = 0.71073 Å
*a* = 10.2656 (10) Å	Cell parameters from 7613 reflections
*b* = 11.0655 (11) Å	θ = 2.5–34.3º
*c* = 11.5029 (12) Å	µ = 0.07 mm^−^^1^
α = 63.646 (6)º	*T* = 150 K
β = 85.669 (6)º	Fragment, colorless
γ = 73.036 (5)º	0.40 × 0.37 × 0.30 mm
*V* = 1117.69 (19) Å^3^	

### Data collection

**Table:**

Nonius KappaCCD diffractometer with Oxford Cryostream	6916 reflections with *I* > 2σ(*I*)
Radiation source: fine-focus sealed tube	*R*_int_ = 0.023
Monochromator: graphite	θ_max_ = 34.3º
*T* = 150 K	θ_min_ = 2.6º
ω and φ scans	*h* = −15→16
Absorption correction: none	*k* = −17→16
35420 measured reflections	*l* = −17→17
8550 independent reflections	

### Refinement

**Table:**

Refinement on *F*^2^	Hydrogen site location: inferred from neighbouring sites
Least-squares matrix: full	H-atom parameters constrained
*R*[*F*^2^ > 2σ(*F*^2^)] = 0.049	*w* = 1/[σ^2^(*F*_o_^2^) + (0.0675*P*)^2^ + 0.2841*P*] where *P* = (*F*_o_^2^ + 2*F*_c_^2^)/3
*wR*(*F*^2^) = 0.142	(Δ/σ)_max_ < 0.001
*S* = 1.03	Δρ_max_ = 0.44 e Å^−^^3^
8550 reflections	Δρ_min_ = −0.24 e Å^−^^3^
262 parameters	Extinction correction: SHELXL, Fc^*^=kFc[1+0.001xFc^2^λ^3^/sin(2θ)]^-1/4^
Primary atom site location: structure-invariant direct methods	Extinction coefficient: 0.016 (5)
Secondary atom site location: difference Fourier map	

### Special details

**Table:**

Geometry. All e.s.d.'s (except the e.s.d. in the dihedral angle between two l.s. planes) are estimated using the full covariance matrix. The cell e.s.d.'s are taken into account individually in the estimation of e.s.d.'s in distances, angles and torsion angles; correlations between e.s.d.'s in cell parameters are only used when they are defined by crystal symmetry. An approximate (isotropic) treatment of cell e.s.d.'s is used for estimating e.s.d.'s involving l.s. planes.
Refinement. Refinement of *F*^2^ against ALL reflections. The weighted *R*-factor *wR* and goodness of fit *S* are based on *F*^2^, conventional *R*-factors *R* are based on *F*, with *F* set to zero for negative *F*^2^. The threshold expression of *F*^2^ > σ(*F*^2^) is used only for calculating *R*-factors(gt) *etc*. and is not relevant to the choice of reflections for refinement. *R*-factors based on *F*^2^ are statistically about twice as large as those based on *F*, and *R*- factors based on ALL data will be even larger.

### Fractional atomic coordinates and isotropic or equivalent isotropic displacement parameters (Å^2^)

**Table:**

	*x*	*y*	*z*	*U*_iso_*/*U*_eq_	
N1	0.57024 (7)	0.44531 (8)	0.71728 (7)	0.02058 (14)	
N2	0.63299 (7)	0.27413 (8)	0.63295 (7)	0.02111 (14)	
N3	0.75731 (7)	0.51591 (8)	0.75233 (8)	0.02095 (14)	
N4	0.85651 (8)	0.16323 (8)	0.60760 (8)	0.02270 (15)	
C1	0.54248 (8)	0.36459 (9)	0.67003 (8)	0.02038 (15)	
C2	0.76639 (8)	0.26219 (9)	0.64044 (8)	0.01867 (14)	
C3	0.80944 (8)	0.35136 (8)	0.67783 (8)	0.01795 (14)	
C4	0.93184 (8)	0.38865 (9)	0.68326 (8)	0.02052 (15)	
C5	0.89619 (9)	0.48788 (9)	0.73001 (9)	0.02223 (16)	
C6	0.70349 (8)	0.43622 (8)	0.71773 (8)	0.01811 (14)	
C7	0.39518 (9)	0.37212 (11)	0.65875 (10)	0.02787 (19)	
H7A	0.3624	0.4225	0.5670	0.042*	
H7B	0.3409	0.4223	0.7063	0.042*	
H7C	0.3861	0.2765	0.6957	0.042*	
C8	1.06921 (9)	0.34405 (11)	0.63522 (10)	0.02737 (18)	
H8A	1.1281	0.2617	0.7068	0.041*	
H8B	1.1107	0.4215	0.6026	0.041*	
H8C	1.0585	0.3198	0.5650	0.041*	
C9	0.98058 (11)	0.56497 (12)	0.75378 (12)	0.0329 (2)	
H9A	1.0761	0.5279	0.7389	0.049*	
H9B	0.9737	0.5521	0.8438	0.049*	
H9C	0.9474	0.6655	0.6943	0.049*	
C10	0.67976 (9)	0.61342 (9)	0.79987 (9)	0.02103 (16)	
C11	0.66155 (10)	0.56408 (10)	0.93308 (9)	0.02507 (17)	
C12	0.57765 (11)	0.65810 (12)	0.97643 (10)	0.0316 (2)	
H12	0.5617	0.6257	1.0662	0.038*	
C13	0.51689 (11)	0.79804 (12)	0.89118 (11)	0.0324 (2)	
C14	0.54134 (11)	0.84480 (10)	0.75999 (11)	0.0306 (2)	
H14	0.5027	0.9412	0.7019	0.037*	
C15	0.62119 (10)	0.75379 (10)	0.71134 (9)	0.02509 (17)	
C16	0.73039 (13)	0.41372 (12)	1.02726 (11)	0.0361 (2)	
H16A	0.8280	0.4010	1.0384	0.054*	
H16B	0.7184	0.3503	0.9933	0.054*	
H16C	0.6896	0.3920	1.1114	0.054*	
C17	0.42260 (16)	0.89708 (17)	0.93916 (17)	0.0527 (4)	
H17A	0.4363	0.8567	1.0339	0.079*	
H17B	0.3277	0.9109	0.9158	0.079*	
H17C	0.4426	0.9880	0.8989	0.079*	
C18	0.63861 (13)	0.80407 (12)	0.56796 (10)	0.0363 (2)	
H18A	0.6251	0.9060	0.5266	0.054*	
H18B	0.5713	0.7828	0.5297	0.054*	
H18C	0.7308	0.7562	0.5541	0.054*	
C19	0.79893 (10)	0.10010 (11)	0.54297 (10)	0.02763 (19)	
H19A	0.7231	0.0670	0.5926	0.033*	
H19B	0.8700	0.0172	0.5434	0.033*	
C20	0.74631 (12)	0.20307 (13)	0.40350 (11)	0.0331 (2)	
H20A	0.8205	0.2380	0.3545	0.050*	
H20B	0.6717	0.2824	0.4029	0.050*	
H20C	0.7129	0.1552	0.3633	0.050*	
C21	0.97891 (9)	0.06971 (10)	0.69446 (10)	0.02640 (18)	
H21A	1.0052	0.1202	0.7372	0.032*	
H21B	1.0547	0.0489	0.6413	0.032*	
C22	0.96184 (11)	−0.06949 (10)	0.79938 (11)	0.0313 (2)	
H22A	0.9405	−0.1225	0.7567	0.038*	
H22B	1.0496	−0.1263	0.8512	0.038*	
C23	0.85034 (12)	−0.05228 (12)	0.89113 (11)	0.0352 (2)	
H23A	0.8677	0.0063	0.9295	0.042*	
H23B	0.7610	−0.0024	0.8409	0.042*	
C24	0.84413 (15)	−0.19306 (14)	0.99966 (13)	0.0455 (3)	
H24A	0.9303	−0.2401	1.0531	0.068*	
H24B	0.8293	−0.2523	0.9621	0.068*	
H24C	0.7688	−0.1775	1.0538	0.068*	

### Atomic displacement parameters (Å^2^)

**Table:**

	*U*^11^	*U*^22^	*U*^33^	*U*^12^	*U*^13^	*U*^23^
N1	0.0157 (3)	0.0244 (3)	0.0239 (3)	−0.0046 (2)	0.0023 (2)	−0.0136 (3)
N2	0.0178 (3)	0.0246 (3)	0.0247 (3)	−0.0066 (3)	0.0031 (2)	−0.0142 (3)
N3	0.0175 (3)	0.0220 (3)	0.0271 (4)	−0.0050 (2)	0.0014 (2)	−0.0146 (3)
N4	0.0213 (3)	0.0227 (3)	0.0268 (4)	−0.0027 (3)	0.0018 (3)	−0.0155 (3)
C1	0.0163 (3)	0.0249 (4)	0.0216 (4)	−0.0061 (3)	0.0024 (3)	−0.0119 (3)
C2	0.0179 (3)	0.0192 (3)	0.0189 (3)	−0.0038 (3)	0.0021 (3)	−0.0096 (3)
C3	0.0152 (3)	0.0190 (3)	0.0194 (3)	−0.0035 (2)	0.0015 (2)	−0.0093 (3)
C4	0.0151 (3)	0.0226 (4)	0.0234 (4)	−0.0045 (3)	0.0017 (3)	−0.0105 (3)
C5	0.0181 (3)	0.0237 (4)	0.0265 (4)	−0.0065 (3)	0.0008 (3)	−0.0120 (3)
C6	0.0164 (3)	0.0189 (3)	0.0198 (3)	−0.0040 (3)	0.0013 (3)	−0.0099 (3)
C7	0.0166 (4)	0.0387 (5)	0.0354 (5)	−0.0096 (3)	0.0040 (3)	−0.0219 (4)
C8	0.0163 (3)	0.0323 (5)	0.0346 (5)	−0.0069 (3)	0.0055 (3)	−0.0165 (4)
C9	0.0258 (4)	0.0348 (5)	0.0481 (6)	−0.0129 (4)	0.0015 (4)	−0.0242 (5)
C10	0.0205 (4)	0.0219 (4)	0.0240 (4)	−0.0047 (3)	0.0012 (3)	−0.0139 (3)
C11	0.0267 (4)	0.0279 (4)	0.0233 (4)	−0.0090 (3)	0.0006 (3)	−0.0128 (3)
C12	0.0343 (5)	0.0409 (5)	0.0287 (5)	−0.0131 (4)	0.0069 (4)	−0.0227 (4)
C13	0.0308 (5)	0.0364 (5)	0.0419 (6)	−0.0084 (4)	0.0066 (4)	−0.0290 (5)
C14	0.0317 (5)	0.0232 (4)	0.0380 (5)	−0.0020 (3)	0.0000 (4)	−0.0180 (4)
C15	0.0274 (4)	0.0222 (4)	0.0259 (4)	−0.0045 (3)	0.0011 (3)	−0.0124 (3)
C16	0.0404 (6)	0.0331 (5)	0.0269 (5)	−0.0091 (4)	−0.0038 (4)	−0.0064 (4)
C17	0.0517 (8)	0.0556 (8)	0.0706 (10)	−0.0091 (6)	0.0167 (7)	−0.0508 (8)
C18	0.0464 (6)	0.0290 (5)	0.0254 (5)	−0.0040 (4)	0.0022 (4)	−0.0094 (4)
C19	0.0304 (4)	0.0284 (4)	0.0321 (5)	−0.0080 (3)	0.0051 (4)	−0.0210 (4)
C20	0.0340 (5)	0.0419 (6)	0.0310 (5)	−0.0103 (4)	0.0019 (4)	−0.0230 (4)
C21	0.0199 (4)	0.0251 (4)	0.0323 (5)	0.0000 (3)	0.0026 (3)	−0.0152 (4)
C22	0.0313 (5)	0.0232 (4)	0.0346 (5)	0.0010 (3)	0.0004 (4)	−0.0138 (4)
C23	0.0371 (5)	0.0291 (5)	0.0316 (5)	−0.0034 (4)	0.0041 (4)	−0.0107 (4)
C24	0.0464 (7)	0.0379 (6)	0.0376 (6)	−0.0104 (5)	0.0024 (5)	−0.0050 (5)

### Geometric parameters (Å, °)

**Table:**

N1—C1	1.3332 (11)	C13—C14	1.3904 (16)
N1—C6	1.3422 (11)	C13—C17	1.5100 (15)
N2—C2	1.3425 (11)	C14—C15	1.3959 (13)
N2—C1	1.3505 (11)	C14—H14	0.9500
N3—C6	1.3676 (11)	C15—C18	1.5035 (15)
N3—C5	1.3972 (11)	C16—H16A	0.9800
N3—C10	1.4331 (11)	C16—H16B	0.9800
N4—C2	1.3860 (10)	C16—H16C	0.9800
N4—C19	1.4692 (12)	C17—H17A	0.9800
N4—C21	1.4700 (12)	C17—H17B	0.9800
C1—C7	1.5023 (12)	C17—H17C	0.9800
C2—C3	1.4190 (11)	C18—H18A	0.9800
C3—C6	1.4095 (11)	C18—H18B	0.9800
C3—C4	1.4458 (11)	C18—H18C	0.9800
C4—C5	1.3743 (12)	C19—C20	1.5207 (16)
C4—C8	1.5020 (12)	C19—H19A	0.9900
C5—C9	1.4923 (13)	C19—H19B	0.9900
C7—H7A	0.9800	C20—H20A	0.9800
C7—H7B	0.9800	C20—H20B	0.9800
C7—H7C	0.9800	C20—H20C	0.9800
C8—H8A	0.9800	C21—C22	1.5280 (15)
C8—H8B	0.9800	C21—H21A	0.9900
C8—H8C	0.9800	C21—H21B	0.9900
C9—H9A	0.9800	C22—C23	1.5261 (16)
C9—H9B	0.9800	C22—H22A	0.9900
C9—H9C	0.9800	C22—H22B	0.9900
C10—C15	1.3980 (13)	C23—C24	1.5194 (17)
C10—C11	1.3981 (13)	C23—H23A	0.9900
C11—C12	1.3949 (14)	C23—H23B	0.9900
C11—C16	1.5068 (15)	C24—H24A	0.9800
C12—C13	1.3890 (17)	C24—H24B	0.9800
C12—H12	0.9500	C24—H24C	0.9800
			
C1—N1—C6	112.50 (7)	C15—C14—H14	119.1
C2—N2—C1	119.06 (7)	C14—C15—C10	117.71 (9)
C6—N3—C5	108.48 (7)	C14—C15—C18	120.94 (9)
C6—N3—C10	124.21 (7)	C10—C15—C18	121.30 (8)
C5—N3—C10	127.29 (7)	C11—C16—H16A	109.5
C2—N4—C19	117.58 (7)	C11—C16—H16B	109.5
C2—N4—C21	118.08 (7)	H16A—C16—H16B	109.5
C19—N4—C21	115.32 (7)	C11—C16—H16C	109.5
N1—C1—N2	127.02 (8)	H16A—C16—H16C	109.5
N1—C1—C7	117.04 (8)	H16B—C16—H16C	109.5
N2—C1—C7	115.94 (8)	C13—C17—H17A	109.5
N2—C2—N4	117.28 (7)	C13—C17—H17B	109.5
N2—C2—C3	119.88 (7)	H17A—C17—H17B	109.5
N4—C2—C3	122.82 (7)	C13—C17—H17C	109.5
C6—C3—C2	113.94 (7)	H17A—C17—H17C	109.5
C6—C3—C4	106.79 (7)	H17B—C17—H17C	109.5
C2—C3—C4	139.21 (8)	C15—C18—H18A	109.5
C5—C4—C3	106.48 (7)	C15—C18—H18B	109.5
C5—C4—C8	124.37 (8)	H18A—C18—H18B	109.5
C3—C4—C8	128.84 (8)	C15—C18—H18C	109.5
C4—C5—N3	109.62 (7)	H18A—C18—H18C	109.5
C4—C5—C9	129.98 (8)	H18B—C18—H18C	109.5
N3—C5—C9	120.38 (8)	N4—C19—C20	112.44 (8)
N1—C6—N3	124.15 (7)	N4—C19—H19A	109.1
N1—C6—C3	127.23 (7)	C20—C19—H19A	109.1
N3—C6—C3	108.55 (7)	N4—C19—H19B	109.1
C1—C7—H7A	109.5	C20—C19—H19B	109.1
C1—C7—H7B	109.5	H19A—C19—H19B	107.8
H7A—C7—H7B	109.5	C19—C20—H20A	109.5
C1—C7—H7C	109.5	C19—C20—H20B	109.5
H7A—C7—H7C	109.5	H20A—C20—H20B	109.5
H7B—C7—H7C	109.5	C19—C20—H20C	109.5
C4—C8—H8A	109.5	H20A—C20—H20C	109.5
C4—C8—H8B	109.5	H20B—C20—H20C	109.5
H8A—C8—H8B	109.5	N4—C21—C22	114.50 (8)
C4—C8—H8C	109.5	N4—C21—H21A	108.6
H8A—C8—H8C	109.5	C22—C21—H21A	108.6
H8B—C8—H8C	109.5	N4—C21—H21B	108.6
C5—C9—H9A	109.5	C22—C21—H21B	108.6
C5—C9—H9B	109.5	H21A—C21—H21B	107.6
H9A—C9—H9B	109.5	C23—C22—C21	113.98 (8)
C5—C9—H9C	109.5	C23—C22—H22A	108.8
H9A—C9—H9C	109.5	C21—C22—H22A	108.8
H9B—C9—H9C	109.5	C23—C22—H22B	108.8
C15—C10—C11	122.03 (8)	C21—C22—H22B	108.8
C15—C10—N3	119.07 (8)	H22A—C22—H22B	107.7
C11—C10—N3	118.86 (8)	C24—C23—C22	112.13 (10)
C12—C11—C10	118.08 (9)	C24—C23—H23A	109.2
C12—C11—C16	120.88 (9)	C22—C23—H23A	109.2
C10—C11—C16	121.05 (9)	C24—C23—H23B	109.2
C13—C12—C11	121.45 (10)	C22—C23—H23B	109.2
C13—C12—H12	119.3	H23A—C23—H23B	107.9
C11—C12—H12	119.3	C23—C24—H24A	109.5
C12—C13—C14	118.91 (9)	C23—C24—H24B	109.5
C12—C13—C17	120.70 (11)	H24A—C24—H24B	109.5
C14—C13—C17	120.36 (11)	C23—C24—H24C	109.5
C13—C14—C15	121.74 (9)	H24A—C24—H24C	109.5
C13—C14—H14	119.1	H24B—C24—H24C	109.5
			
C6—N1—C1—N2	4.24 (13)	C10—N3—C6—C3	−179.03 (8)
C6—N1—C1—C7	−177.02 (8)	C2—C3—C6—N1	−3.57 (13)
C2—N2—C1—N1	−1.29 (14)	C4—C3—C6—N1	174.07 (8)
C2—N2—C1—C7	179.97 (8)	C2—C3—C6—N3	179.42 (7)
C1—N2—C2—N4	176.89 (8)	C4—C3—C6—N3	−2.94 (9)
C1—N2—C2—C3	−4.58 (12)	C6—N3—C10—C15	−90.87 (11)
C19—N4—C2—N2	11.95 (12)	C5—N3—C10—C15	87.33 (11)
C21—N4—C2—N2	−133.70 (9)	C6—N3—C10—C11	87.18 (11)
C19—N4—C2—C3	−166.54 (8)	C5—N3—C10—C11	−94.62 (11)
C21—N4—C2—C3	47.81 (12)	C15—C10—C11—C12	2.55 (14)
N2—C2—C3—C6	6.56 (12)	N3—C10—C11—C12	−175.43 (8)
N4—C2—C3—C6	−174.99 (8)	C15—C10—C11—C16	−177.49 (9)
N2—C2—C3—C4	−169.98 (10)	N3—C10—C11—C16	4.53 (13)
N4—C2—C3—C4	8.47 (16)	C10—C11—C12—C13	−2.01 (15)
C6—C3—C4—C5	2.29 (9)	C16—C11—C12—C13	178.03 (10)
C2—C3—C4—C5	178.98 (10)	C11—C12—C13—C14	−0.34 (16)
C6—C3—C4—C8	−171.43 (9)	C11—C12—C13—C17	177.93 (11)
C2—C3—C4—C8	5.26 (17)	C12—C13—C14—C15	2.29 (16)
C3—C4—C5—N3	−0.82 (10)	C17—C13—C14—C15	−175.98 (11)
C8—C4—C5—N3	173.25 (8)	C13—C14—C15—C10	−1.77 (15)
C3—C4—C5—C9	−179.00 (10)	C13—C14—C15—C18	175.79 (11)
C8—C4—C5—C9	−4.92 (16)	C11—C10—C15—C14	−0.71 (14)
C6—N3—C5—C4	−1.01 (10)	N3—C10—C15—C14	177.27 (8)
C10—N3—C5—C4	−179.44 (8)	C11—C10—C15—C18	−178.26 (10)
C6—N3—C5—C9	177.37 (9)	N3—C10—C15—C18	−0.28 (14)
C10—N3—C5—C9	−1.06 (14)	C2—N4—C19—C20	71.72 (11)
C1—N1—C6—N3	175.04 (8)	C21—N4—C19—C20	−141.70 (9)
C1—N1—C6—C3	−1.54 (13)	C2—N4—C21—C22	91.90 (10)
C5—N3—C6—N1	−174.64 (8)	C19—N4—C21—C22	−54.51 (11)
C10—N3—C6—N1	3.84 (14)	N4—C21—C22—C23	−60.09 (12)
C5—N3—C6—C3	2.48 (10)	C21—C22—C23—C24	−175.74 (10)
